# The effect of low-temperature annealing on discordance of U–Pb zircon ages

**DOI:** 10.1038/s41598-021-86449-y

**Published:** 2021-03-29

**Authors:** Maria Herrmann, Ulf Söderlund, Anders Scherstén, Tomas Næraa, Sanna Holm-Alwmark, Carl Alwmark

**Affiliations:** 1 Department of Geology, Lund University, Sölvegatan 12, 22362 Lund, Sweden; 2 Department of Geosciences, Swedish Museum of Natural History, Box 50007, 10405 Stockholm, Sweden; 3 Niels-Bohr Institute, University of Copenhagen, Copenhagen, Denmark; 4 Natural History Museum Denmark, University of Copenhagen, Copenhagen, Denmark

**Keywords:** Planetary science, Solid Earth sciences

## Abstract

Discordant U–Pb data of zircon are commonly attributed to Pb loss from domains with variable degree of radiation damage that resulted from α-decay of U and Th, which often complicates the correct age interpretation of the sample. Here we present U–Pb zircon data from 23 samples of ca. 1.7–1.9 Ga granitoid rocks in and around the Siljan impact structure in central Sweden. Our results show that zircon from rocks within the structure that form an uplifted central plateau lost significantly less radiogenic Pb compared to zircon grains in rocks outside the plateau. We hypothesize that zircon in rocks within the central plateau remained crystalline through continuous annealing of crystal structure damages induced from decay of U and Th until uplifted to the surface by the impact event ca. 380 Ma ago. In contrast, zircon grains distal to the impact have accumulated radiation damage at shallow and cool conditions since at least 1.26 Ga, making them vulnerable to fluid-induced Pb-loss. Our data are consistent with studies on alpha recoil and fission tracks, showing that annealing in zircon occurs at temperatures as low as 200–250 °C. Zircon grains from these samples are texturally simple, i.e., neither xenocrysts nor metamorphic overgrowths have been observed. Therefore, the lower intercepts obtained from regression of variably discordant zircon data are more likely recording the age of fluid-assisted Pb-loss from radiation-damaged zircon at shallow levels rather than linked to regional magmatic or tectonic events.

## Introduction

The U–Pb decay system in zircon is the most widely used geochronometer for the determination of radiometric ages, due to its general robustness in geological systems and the direct control of closed-system behaviour through the dual decay of uranium. In addition, analytical techniques such as Secondary Ion Mass Spectrometry (SIMS) and Laser Ablation-Inductive Coupled Plasma-Mass Spectrometry (LA-ICP-MS) enables high-spatial resolution analysis and age determination of multiple discrete events from a single zircon grain e.g.^1,2^.

Accumulation of radiogenic Pb over time is linked to the ^238^U-^206^Pb, ^235^U-^207^Pb and ^232^Th-^208^Pb decay chains, which is associated with recoil during emission of alpha particles and radiation damage e.g.^3^. Despite the robustness of the U–Pb system in zircon, concordant data are relatively rare even in pristine igneous rocks^4^. The geological cause of discordance is generally attributed to the loss of radiogenic Pb through solid-state diffusion, fluid-assisted element mobility in radiation-damaged zircon, or recrystallization of metamict domains^5^ (and references therein). Solid-state diffusional Pb-loss from crystalline zircon requires temperatures of at least 900−1000 °C^6,7^. Loss at significantly lower temperatures occurs more readily in radiation-damaged zircon, especially in the presence of fluids^8–11^.

Experimental studies show that radiation-damaged zircon is able to recover through annealing when the temperature is sufficiently high. Depending on the amount of radiation damage, the annealing temperature in highly damaged zircon on short timescales (minutes to days) has been estimated to 850 and 1200 °C under dry conditions^12,13^ which exceed those of the deep crust and silicic magmas. However, over geological time scales, structural defects, such as alpha damage (point defects and recoil tracks^14^) and fission tracks, anneal at significantly lower temperatures although estimates vary. Annealing of fission tracks have been suggested to occur at temperatures between 200 and 250°C^15–17^ while alpha damage anneals over a broader temperature range^14,18^. This leads to the hypothesis that Pb-loss is enhanced at shallow, relatively cool, crustal levels due to the accumulated effects of radiation damage and fluid-assisted element mobility, as suggested by^19^. However, this hypothesis has yet to be empirically tested on natural zircon.

**Figure 1:**
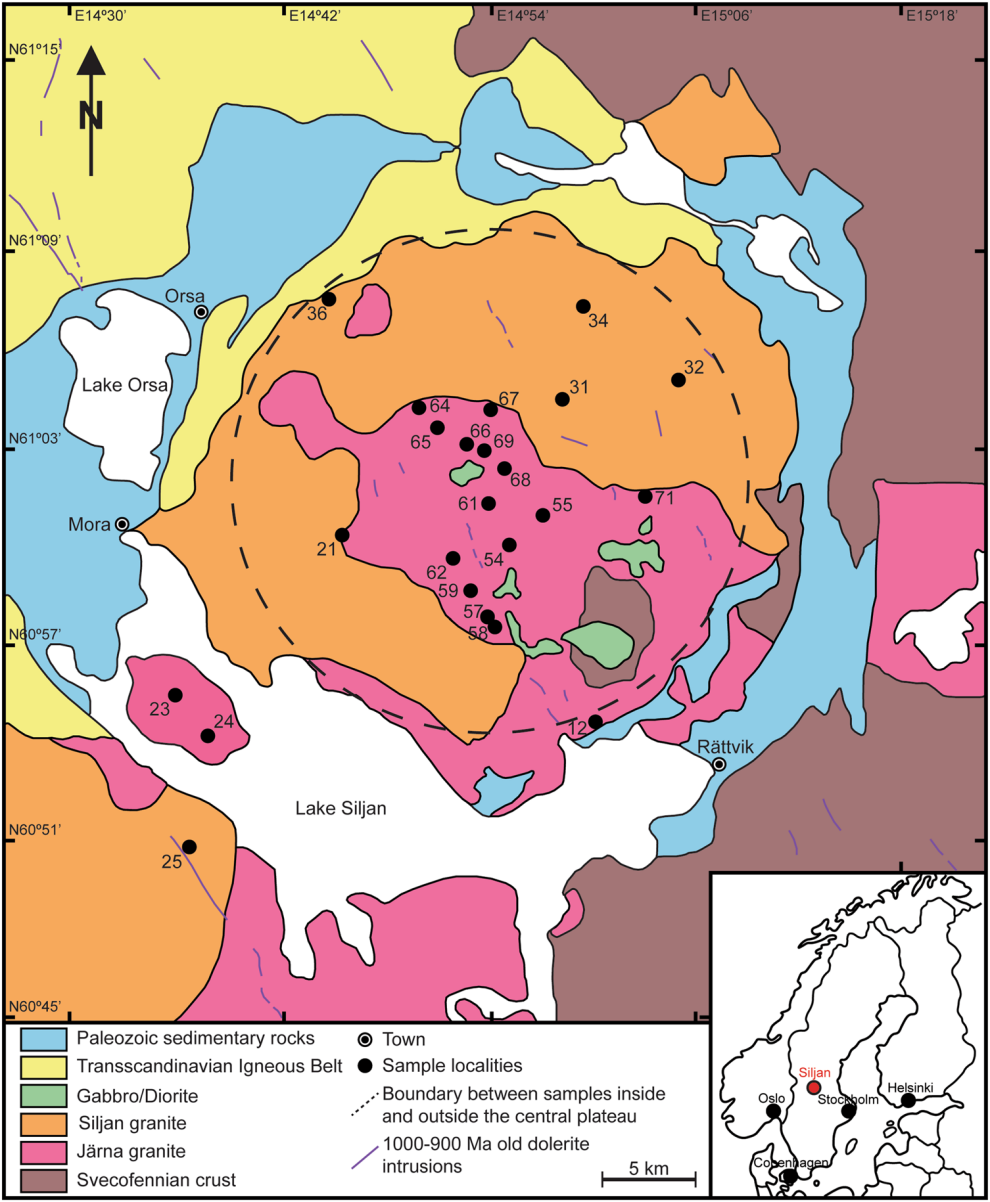
Geological map modified after^21^ of the Siljan impact structure. Sample localities of Järna and Siljan granite inside and outside the central plateau are shown. The inset map shows the location of the Siljan impact structure in Sweden (red point). (Map modified with Adobe Illustrator version v. 25.0.1, https://www.adobe.com/products/illustrator.html?promoid=PGRQQLFS&mv=other#).

We have analysed the U–Pb systematics in zircon from 23 near-surface samples of Paleoproterozoic basement rocks in and around the Siljan impact structure in northern Dalarna, central Sweden (Fig. 1), using LA-ICP-MS, supported by Field-Emission Scanning Electron Microscopy (FE-SEM). The Siljan impact structure is located at the boundary between the Transscandinavian Igneous Belt (TIB) and the westernmost domain of the Svecofennian crust (Fig. 1). The structure has an estimated rim-to-rim diameter of about 52 km and a 28–30 km wide central plateau that is dominated by Järna and Siljan granite^20,21^ (Fig. 1).Traditionally both granite types are assigned to the 1.81–1.65 Ga-old TIB rocks^22^. However, a recent re-organization of the lithotectonic framework of Sweden by^23^ refers Järna granite to syn-Svecokarelian rocks, formerly addressed as Svecofennian crust^23^, whilst Siljan granite builds part of the post-Svecokarelian rocks. The central plateau is surrounded by an annular depression that is partly filled by lakes and down-faulted Paleozoic sediments^24–27^ (Fig. 1).

Zircon U–Pb crystallization ages of Järna and Siljan granite range between 1.9 and 1.7 Ga^28–30^. In northern Dalarna where the Siljan impact structure is located, dolerite dykes and sills have yielded concordant to near concordant U–Pb baddeleyite ages that fall in the 1462–1461 Ma, 1271–1264, and 978–946 Ma age intervals^31^. Previous K–Ar whole rock ages^32^ and Rb–Sr whole rock-biotite dates^33^ performed on the two younger generations of dolerite yielded similar age estimates. These mafic rocks are pristine with no signs of post-magmatic metamorphism or fluid-induced alteration. Considering that both the Rb–Sr system and baddeleyite are sensitive to such events (e.g.^34^) in conjunction with the general low closure temperature for the isotopic K–Ar system (250–550°C^35,36^), there cannot have been significant thermal heating or fluid activity in the Siljan region after at least ca. 1260 Ma until the impact event occurred which has been dated at 380.9 ± 4.6 Ma through ^40^Ar/^39^Ar on melt dikelets and melt breccias^37,38^ Dolerite dykes and sills that intruded the Siljan region about 1000–900 Ma^32^ are scarce (Fig. 1) and could only have caused local heating of host rocks in the immediate contact to the intrusions. In general, the TIB plutonic rocks in Sweden are associated with abundant volcanic rocks of the same age, which in Dalarna are represented by the so-called Dala porphyries and ignimbrites (~ 1.8 to ~ 1.7 Ga, e.g.^39^) that are abundant immediately northwest of Siljan^22^. The co-existence of plutonic and volcanic rocks at present-day erosional surface suggest relatively shallow crystallization of the Järna and Siljan granites and only moderate erosion. Based on zircon and titanite fission track data with dates between ~ 900 and ~ 800 Ma, a sediment cover derived from eroding Caledonides of about 2 km has been suggested at the time of impact^40,41^. However, the existence of such a former thick sedimentary sheet throughout Sweden was questioned by^42^. Since the impact event, the Siljan impact structure underwent erosion of about 4 km^27^ without further tectonic or magmatic activities.

Based on planar deformation features in quartz and occurrences of shatter cones from inside the central plateau, the amount of uplift during the impact event is modelled to about 8 km^27,43^ with calculated shock pressures from 2 to 16 GPa^21,43^ for the rocks inside the central plateau. In contrast, the basement rocks outside the central plateau resided at or near current erosional surface since at least 1260 Ma^44,45^, and presumably as early as the TIB crystallized. Assuming a thick sedimentary cover existed, the zircon and titanite fission track data by^41^ would support this hypothesis and would also suggest that the basement rock outside the central plateau resided near-surface since ~ 800–900 Ma. Given an ancient geothermal gradient of 29–32 °C/km for the granites in Siljan prior to the impact event^46^, the rocks inside the central plateau resided near the zircon annealing temperature until the impact-induced uplift. If zircon indeed anneal around 250 °C over geological time scales, the zircon from the central plateau should be less discordant than those from outside the central plateau.

## Results

The investigated rocks of Siljan and Järna type are reddish, medium to coarse-grained, with equigranular to porphyritic textures. Rocks inside the central plateau are significantly affected by hydrothermal alteration, with a decrease of alteration away from the centre. Further details on samples are provided in the Supplementary Figure S1.

FE-SEM imaging and qualitative element analyses show that zircon grains from samples outside the central plateau more commonly have metamict domains (about 45% of the dated grains) with higher concentrations of non-stochiometric elements (e.g., Na, Ca, Fe) compared to zircon grains within the central plateau where about 6% of the dated grains show textural evidence for metamictization (see Supplementary Fig. S2 and S3). Zircon and quartz from the central plateau display planar features, that are exclusively impact-generated^47–49^ (see Supplementary Fig. S1 and S2). No shock-metamorphic features have been found in zircon or quartz from rocks outside the central plateau.

**Figure 2:**
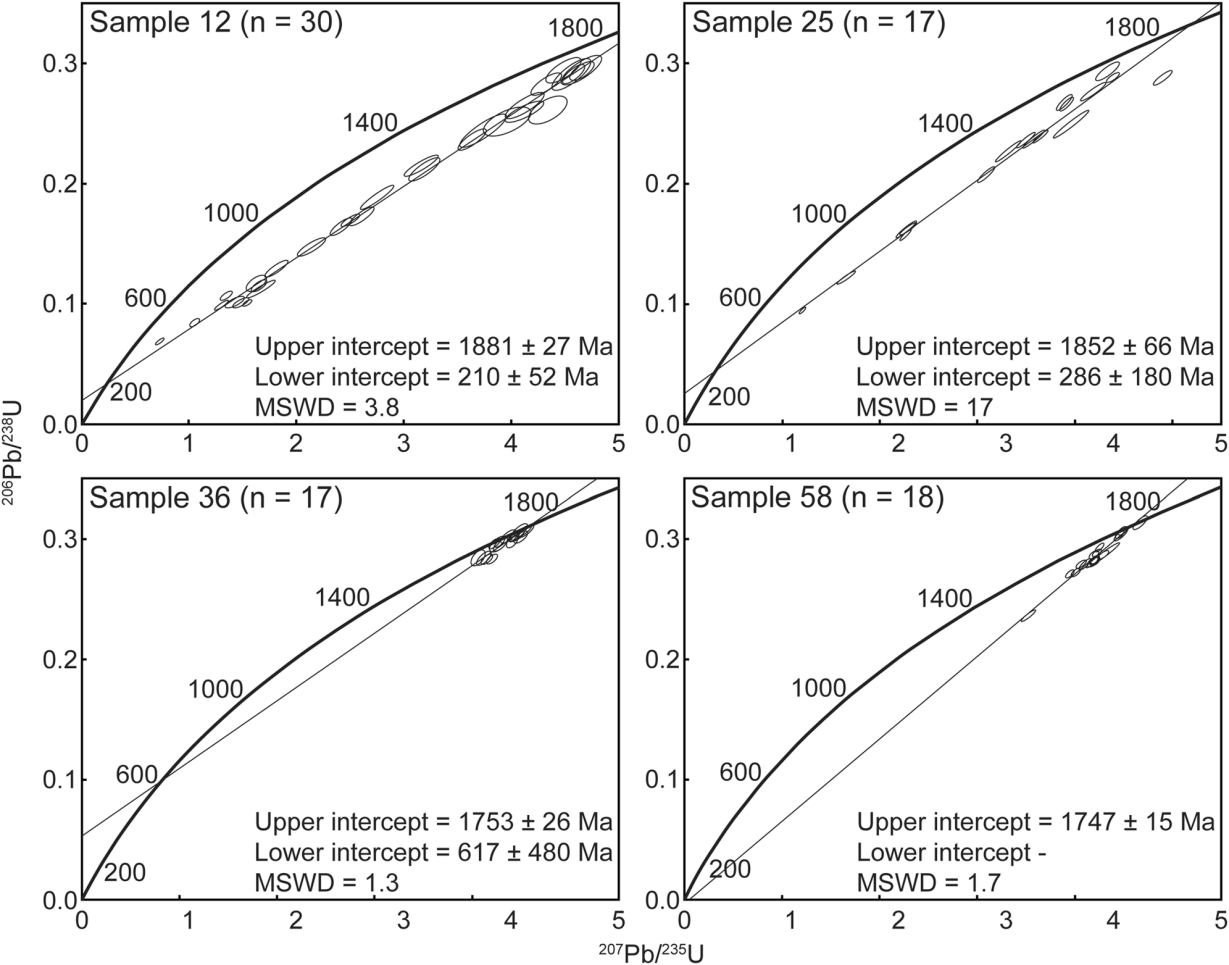
Representative concordia plots for samples outside the central plateau (samples 12 = Järna granite and 25 = Siljan granite) and inside the central plateau (samples 36 = Siljan granite and 58 = Järna granite). (Plots created with with Adobe Illustrator version v. 25.0.1, https://www.adobe.com/products/illustrator.html?promoid=PGRQQLFS&mv=other#).

The U–Pb LA-ICP-MS data are presented in Table 1 as well as in Supplementary Table S1 and Supplementary Figure S4. Supplementary Table S2 gives the U–Pb LA-ICP-MS data of the used references materials. A representative summary of concordia plots are given in Fig. 2. Data quality was evaluated using the following criteria: (1) signal duration longer than 5 s, (2) spot location exclusively within single zircon domains with no portion of epoxy, (3) concordant or normally discordant data, and (4) data with no detectable common Pb. Data was screened for common Pb through plotting ^206^Pb/^204^Pb against the ^207^Pb/^206^Pb. Data with a significant increase in the ^207^Pb/^206^Pb defines the lower limit of ^206^Pb/^204^Pb and was culled (e.g., see Supplementary Fig. S5). The lower limit of ^206^Pb/^204^Pb varies between the samples but commonly range between 10^3^ and 10^4^. Spot analyses that do not match the criteria were rejected from the age calculations.

**Table 1:** Summary of the U–Pb data, including the upper and lower intercept dates, MSWD, n = number of analyses, coordinates of sample localities and granite type.

Sample number	Lower intercept age (Ma)	Upper intercept age (Ma)	MSWD	n	Location		Description
					Lat (°N)	Long (°E)	
**Outside central plateau**							
12	210 ± 52	1881 ± 27	3.80	30	60° 54.503′	15° 00.197′	Järna granite
23	198 ± 170	1833 ± 29	7.90	32	60° 54.964′	14° 37.008′	Järna granite
24	255 ± 62	1804 ± 15	2.30	27	60° 53.896′	14° 38.071′	Järna granite
25	286 ± 180	1852 ± 66	17.00	17	60° 51.087′	14° 36.016′	Siljan granite
**Inside central plateau**							
21	106 ± 350	1815 ± 21	8.70	23	60° 59.569′	14° 46.760′	Siljan granite
31	162 ± 190	1773 ± 33	3.50	20	61° 04.258′	14° 57.545′	Siljan granite
32	554 ± 1100	1756 ± 35	2.20	9	61° 05.236′	15° 02.643′	Siljan granite
34	128 ± 400	1747 ± 13	1.30	11	61° 07.016′	14° 59.630′	Siljan granite
36	617 ± 480	1753 ± 26	1.30	17	61° 07.133′	14° 48.314′	Siljan granite
54	–	1733 ± 19	2.80	38	61° 00.117′	14° 54.557′	Järna granite
55	–	1701 ± 17	0.77	17	61° 00.885′	14° 53.894′	Järna granite
57	67 ± 360	1736 ± 19	1.40	18	60° 57.721′	14° 53.894′	Järna granite
58	–	1747 ± 15	1.70	18	60° 57.409′	14° 54.355′	Järna granite
59	–	1805 ± 16	0.92	8	60° 58.524′	14° 52.866′	Järna granite
61	11 ± 480	1752 ± 20	2.10	26	61° 01.268′	14° 53.411′	Järna granite
62b	7 ± 340	1727.3 ± 5.8	1.09	21	60° 59.524′	14° 52.030′	Järna granite
64a	65 ± 350	1783 ± 10	0.60	14	61° 04.046′	14° 50.075′	Järna granite
65	321 ± 1100	1815 ± 28	2.90	6	61° 03.671′	14° 50.729′	Järna granite
66	426 ± 280	1727 ± 29	2.10	8	61° 03.190′	14° 52.150′	Järna granite
67	–	1713 ± 18	1.20	12	61° 04.146′	14° 53.946′	Järna granite
68	–	1714.0 ± 9.4	1.60	20	61° 02.111′	14° 54.790′	Järna granite
69	37 ± 680	1726 ± 14	0.69	14	61° 03.051′	14° 53.612′	Järna granite
71	–	1714 ± 28	0.40	12	61° 01.604′	15° 02.803′	Järna granite

Data was plotted in Wetherill concordia diagrams and linear regressions were performed of each population, revealing a range of concordant to discordant analyses (see Supplementary Fig. S4). The regression typically yielded mean square weighted deviates (MSWD) between 0.4 and 17.0. Upper intercept dates fall between 1.9 and 1.7 Ga, which corresponds with the known age range of the Siljan and Järna granites^28–30^. Lower intercepts are < 617 Ma with no systematic variation between rocks from inside or outside of the central plateau (see Supplementary Fig. S6).

## Discussion

### The effect of the Siljan impact event on the zircon U–Pb system

It has been shown that impact events can successfully be dated by U–Pb of so-called neoblastic zircon, which preferentially occur in impact melt rocks e.g.^50,51^. Zircon neoblasts tend to form at sites with crystal structure defects (e.g., radiation-damaged domains) at temperatures between 1100 and 1200 °C^48,49,52^. This typically results in strongly discordant data sets for which the lower intercept ideally represents the age of the impact e.g.^53^. Although the 380.9 ± 4.6 Ma age of the Siljan impact event falls within our lower intercept range of ~ 617 to 7 Ma (see Supplementary Fig. S6), textural features indicative for recrystallization into neoblastic zircon is absent in our samples. Planar features, formed at T < 900 °C and P < 20 GPa^49^, suggest that the temperatures were insufficient to cause any resetting of the U–Pb system. As shown in previous studies, the U–Pb system through diffusional or recrystallization processes remains unaffected in zircon from the target rocks at shock pressures up to 20 GPa e.g.^54,55^.

**Figure 3:**
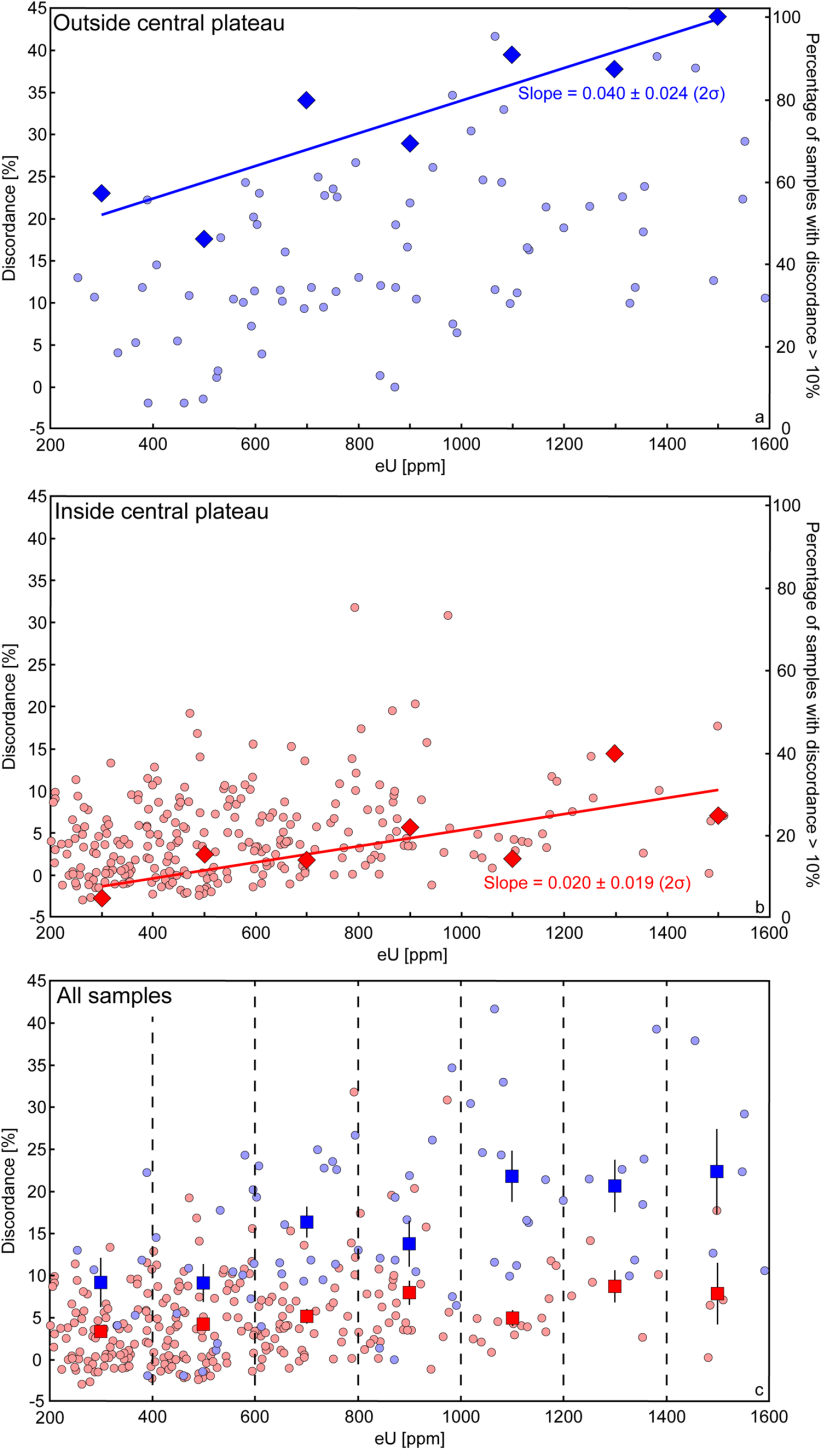
Plots showing the discordance [%] (left y-axis) vs. eU concentration [ppm] of zircon grains (**a**) outside the central plateau, and (**b**) inside the central plateau. Only grains with eU contents between 200 and 1600 ppm are included. In (**a**) and (**b**) both subsets are positively correlated, respectively. The slopes are calculated for the percentage of samples with more than 10% discordance (right y-axis and diamond-shaped data points), showing that the slopes for samples outside and inside the central plateau are 0.040 ± 0.024 (2σ) and 0.020 ± 0.019 (2σ) respectively. In diagram (**c**) both subsets are plotted together with discordance [%] vs. eU [ppm]. Plot (**c**) shows the incremental mean discordances for samples outside (blue squares) and inside (red squares) the central plateau for different eU concentration bins (200–400, 400–600, 600–800, 800–1000, 1000–1200, 1200–1400, and 1400–1600 ppm respectively and highlighted by the vertical dotted lines). Error bars are shown at ± 1 S.E. In each eU concentration bin, the incremental mean discordance of samples from outside the central plateaus is significantly higher than inside. Number of analyses included in the calculation of 1 S.E., and 1 S.D. of the mean discordance for each eU concentration bin are given in Table S3 (plots created with Adobe Illustrator version v. 25.0.1, https://www.adobe.com/products/illustrator.html?promoid=PGRQQLFS&mv=other#).

We have divided the zircon grains into two subsets, one comprising zircon in rocks from outisde the central plateau (Fig. 3a) and one comprising zircon in rocks inside the central plateau (Fig. 3b). Impact-generated hydrothermal activity that might enhance Pb-loss, especially in radiation-damaged zircon, was more prevalent inside the central plateau than outside. It could thus be expected that resetting of zircon within the central plateau should be more effective than outside, and that the lower intercept of these crystals could approximate the age of the impact event. However, there is no significant age difference of the lower intercept dates between the two subsets (see Supplementary Fig. S6). In addition, a majority of the lower intercepts are within the same 150–500 Ma age range as obtained from Paleoproterozoic rocks elsewhere in the Baltic Shield^56^, rendering the lower intercept dates meaningless with respect to the age of the impact. We conclude that the zircon U–Pb system was not significantly affected by the Siljan impact event, or by impact-generated hydrothermal activity.

### The effect of annealing on discordance in zircon

**Figure 4:**
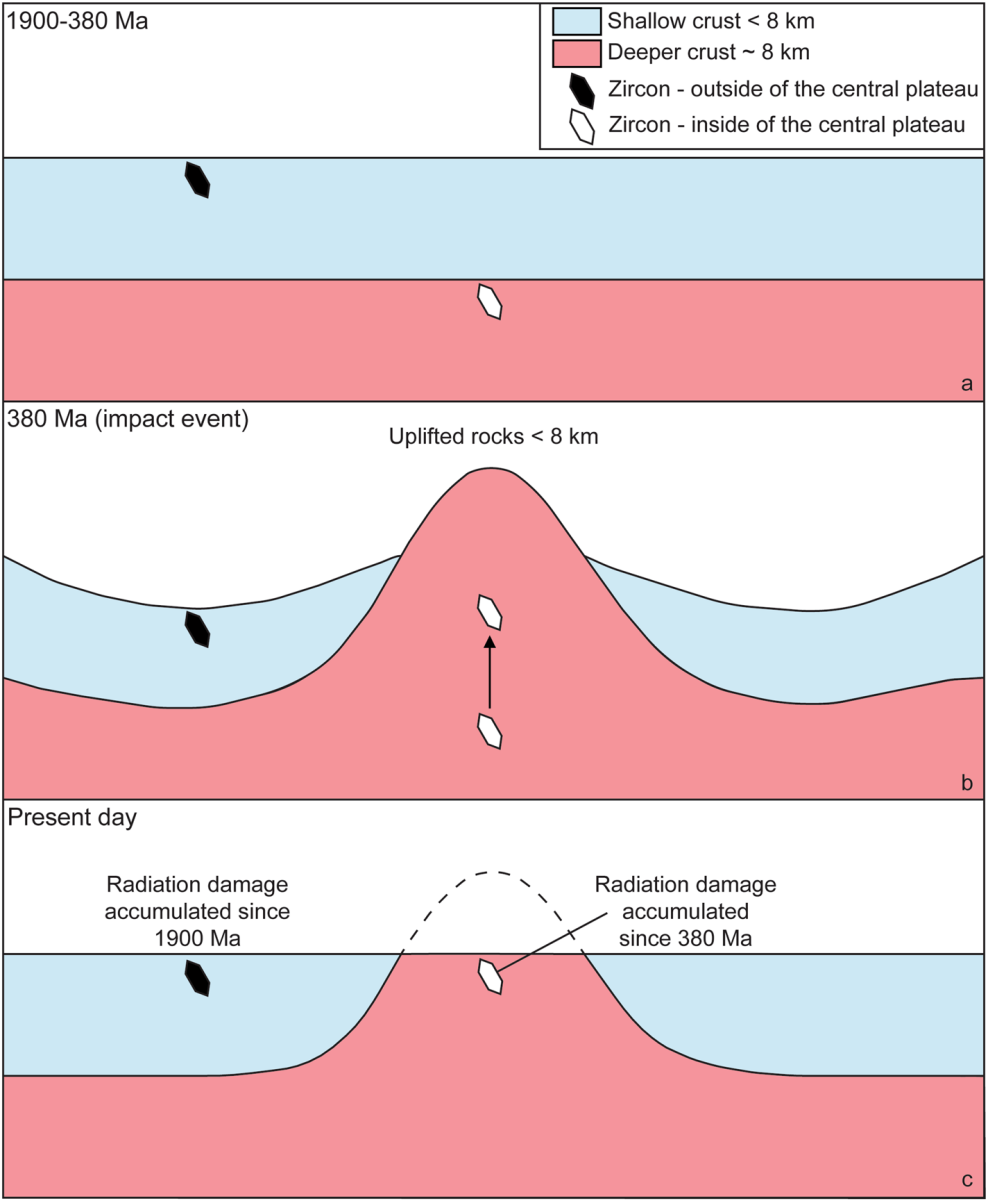
Cross-sections illustrating the evolution of the Siljan area prior (**a**), during (**b**) and after (**c**) the impact event. (**a**) Illustrates the crust in the period 1900–380 Ma. The different depth of zircon outside (black grain) and inside (white grain) the central plateau is shown. The zircon outside the central plateau resided near the surface where radiation damage accumulated since at least 1260 Ma. Zircon inside the central plateau resided at ~ 8 km depth and annealed continuously. (**b**) The impact event caused ~ 8 km uplift of the central plateau, exposing zircon to surface conditions since 380 Ma. (**c**) Present situation, zircon from both out- and inside the central plateau resides at surface (figure created with Adobe Illustrator version v. 25.0.1, https://www.adobe.com/products/illustrator.html?promoid=PGRQQLFS&mv=other#).

We assess the cause and difference of the observed discordance in zircon through plotting the U concentration against the degree of discordance (Fig. 3c). In order to obtain overlapping data for the two subsets, we have screened the effective U (eU) concentration to between 200 and 1600 ppm U. If discordance was entirely controlled by the U concentration, there should be a simple positive linear correlation with no discernible difference between the subsets. However, least square regressions using IsoplotR^57^ of the respective subsets, which include samples with more than 10% discordance, yield different slopes. Zircon populations of samples from outside and inside the central plateau yield slope values of 0.040 ± 0.024 (2σ) and 0.020 ± 0.019 (2σ), respectively (Fig. 3a,b). This implies a greater discordance for the samples outside the central plateau for any given U concentration. To further test this observation, we calculated the mean discordance of the eU concentration intervals 200–400, 400–600, 600–800, 800–1000, 1000–1200, 1200–1400, and 1400–1600 ppm. The difference in the discordance remains regardless of the uranium concentration as shown in Fig. 3c. The mean discordance for samples outside the central plateau increases for each eU concentration interval, from 9.1 ± 2.9 (1 standard error = S.E.)/7.8 (1 standard deviation = S.D.) % at 200–400 ppm eU to 22.5 ± 5.1 (1 S.E.)/11.4 (1 S.D.) % at 1400–1600 ppm eU, while those from inside the central plateau increases from 3.2 ± 0.4 (1 S.E.)/3.8 (1 S.D.) % at 200–400 ppm eU to 7.8 ± 3.6 (1 S.E.)/7.3 (1 S.D.) % at 1400–1600 ppm eU. It is noteworthy that the standard errors of the mean discordance for each eU concentration interval are greater for the subset from outside the central plateau than inside (Fig. 3c). Number of analyses included in the calculation of 1 S.E. and 1 S.D. of the mean discordance for each eU concentration interval can be found in Table S3. A summary of the geological evolution of the Siljan impact structure is shown in Fig. 4. Given that the impact event had no effect on the U–Pb system of the Siljan zircon populations, the most obvious difference between the two subsets are the contrasting residence times at shallow crustal depth. The rocks outside the central plateau have been near the current erosional surface for at least 1260 Ma^44,45^, while the rocks inside the central plateau have been at corresponding shallow level since the time of the impact at 380 Ma (Fig. 4). The contrasting surface residence times might explain the observed difference in discordance between the two subsets if the temperature was sufficiently high for annealing of zircon prior to the uplift of the central plateau. A geothermal gradient of 29–32 °C/km, correspond to a temperature of 256–232 °C for 8 km uplift^46^, which is considerably lower than the annealing temperatures of 600–650 °C postulated by^19^. However, the latter temperature estimates rely on annealing of already radiation-damaged zircon during metamorphism or experiments of short duration, which are not applicable in the present context. It is known that the annealing temperature of fission tracks in zircon is estimated to between 200 and 250 °C^15–17^, whereas annealing of alpha damage may require slightly higher temperatures (> 250 °C^14^) although lower estimates have also been suggested (100–160 °C^18^). With respect to these annealing temperature estimates, we find a remarkably good agreement with the temperature estimates for the uplifted central part of the Siljan structure, which is in consistency with the observed difference in discordance between the two subsets. Our results suggest that annealing of alpha recoil and fission tracks in zircon from Siljan were operating at 200–250 °C, prohibiting accumulation of radiation damage in zircon and hence significant Pb-loss prior to uplift.

In general, Pb loss from radiation-damaged zircon is more efficient in the presence of fluids e.g.^58,59^. It is notable that rocks from the central plateau were heavily fractured and affected by hydrothermal activity due to the impact event^46^ (see Supplementary Fig. S1). This is in stark contrast to the rocks outside the central plateau, which were significantly less affected. Zircon from inside the central plateau should thus have been more affected by fluid interaction and Pb loss. This paradox can be explained if zircon inside the central plateau continuously underwent annealing prior to uplift (Fig. 4), which implies higher crystallinity and less vulnerability to hydrothermal alteration. We conclude that the zircon from the central plateau remained crystalline until uplifted and that surface exposure time remains the single most important factor in explaining the difference in discordance. Continuous Pb-loss near the surface will, with time, lead to excess scatter of analyses around the discordia and also explains the great variance for the subset outside the central plateau. Therefore, the lower intercepts obtained from regression of variably discordant zircon data are more likely recording the age of fluid-assisted Pb-loss from radiation-damaged zircon at shallow levels rather than linked to regional magmatic or tectonic events.

## Methods

Between 45 and 60 zircon grains were separated from each sample for LA-ICP-MS. All preparation and analyses were done at the Department of Geology, Lund University, Sweden. The zircon grains were separated through crushing, sieving, water-based density separation (Wifley Table), hand-picking (100–200 grains per sample) with final size fraction of 80–250 µm, before being mounted in epoxy resin and polished until the central part of each grain was exposed. The zircon crystals are translucent with pinkish or yellowish colour to colourless, and mostly of prismatic and euhedral morphology as well as some rounded grains (see Supplementary Fig. S2).

The FE-SEM imaging was done on a Tescan Mira3 High Resolution Schottky FE-SEM equipped with an Oxford energy dispersive spectrometry (EDS) and a CL system. The accelerating voltage was set to 15 kV. The working distance ranged from 5 to 15 mm. Back-scatter electron (BSE) and cathodoluminescence (CL) of polished zircon crystals was used to guide the spot selection. Primarily, areas that occur homogeneous in BSE and CL were selected.

The LA-ICP-MS (n = 1284 spots) analyses were done using a Bruker Aurora Elite ICP-MS connected to a 193 nm Cetac Analyte G2 excimer laser. The running conditions are summarized in Supplementary Table S4. The repetition rate of the laser was set to 7–8 Hz, a square spot geometry with a size from 18 × 18 µm to 20 × 20 µm was used (area fixed in each sequence), and the fluence was set to ~ 5–6 J/cm^2^. Samples and reference materials were placed in a two volume HelEx2 sample cell flushed with helium gas which was mixed with Ar and N_2_ gas before entering the torch. NIST612 was used to tune the instrument, focusing on stable signals, low oxide production (< 0.5%), Th/U around 1, and high ^207^Pb and ^238^U signals. GJ-1^60^ and 91500^61^ served as primary and secondary reference material, respectively. The analytical sequence was done in automatic mode with a setup starting with 6 GJ-1, followed by analysing 10 unknowns and 3 GJ-1, with up to 100–120 analyses per sequence. The masses ^202^Hg, ^204^(Pb, Hg), ^206^Pb, ^208^Pb, ^232^Th, and ^238^U (in some of the sequences ^202^Hg was left out) were measured in dynamic mode within a single collector system. Dwell times on each mass are listed in Supplementary Table S4. Raw data reduction, including base-line subtraction, drift correction and down-hole correction, was carried out with Iolite^62^ (version 3.63). The construction of the Wetherill concordia plots and age calculations was done by Isoplot Excel Add-in^63^.

## Supplementary Information

Supplementary Information 1.

Supplementary Information 2.

Supplementary Information 3.

Supplementary Information 4.

Supplementary Information 5.
