# Supplementary material for: The effect of low-temperature annealing on discordance of U–Pb zircon ages
Source: Sci Rep. 2021 Mar 29;11:7079. doi: 10.1038/s41598-021-86449-y (PMC8007740; doi:10.1038/s41598-021-86449-y)
Supplement: Supplementary file 1 — Supplementary Information 1. [file 41598_2021_86449_MOESM1_ESM.docx]

Supplementary

**The effect of low-temperature annealing on discordance of U-Pb zircon ages**

**Maria Herrmann^1^*, Ulf Söderlund^1,2^, Anders Scherstén^1^, Tomas Næraa^1^, Sanna Holm-Alwmark^1,3,4^ and Carl Alwmark^1^**

^1^Department of Geology, Lund University, Sölvegatan 12, SE-22362 Lund, Sweden

^2^Department of Geosciences, Swedish Museum of Natural History, Box 50007, SE-10405, Stockholm

^3^Niels-Bohr Institute, University of Copenhagen, Copenhagen, Denmark

^4^Natural History Museum Denmark, University of Copenhagen, Copenhagen, Denmark

*Corresponding author (Maria.[Herrmann@geol.lu.se](mailto:Herrmann@geol.lu.se))

**Content**

Additional information on results

**Additional information on results**

The main minerals are quartz, K-feldspar, plagioclase, biotite, chlorite, amphibole, and to lesser degree, white mica, as well as zircon, titanite, apatite, epidote and opaque minerals as accessory phases. Rocks inside the central plateau have been affected by hydrothermal alteration, which become less away from the centre. Altered K-feldspar and plagioclase are turbid, greyish-brownish in plane-polarized light. The plagioclase core is altered to sericite, while the outer rim is colourless and pristine (Fig. S1a). K-feldspar show perthitic exsolution lamellae of plagioclase (Fig. S1b). Most of the biotite inside the central platau is altered to chlorite showing interference colours from yellow of lower order to anomalous blue in cross-polarized light (Fig. S1c). Less-altered plagioclase and K-feldspar from outside the central plateau are colourless in plane-polarized light with more pronounced grey interference colour in cross-polarized light. Biotite grains are less chloritized, with a strong brownish colour in plane-polarized light (Fig. S1d). Many samples from inside the central plateau are strongly fractured, and show shock features, e.g., kink bands in biotite/chlorite, PDFs in quartz, and ladder textures in K-feldspar (Fig. S1c, e, f). These features occur only in the central localities, where shock pressures were significant.


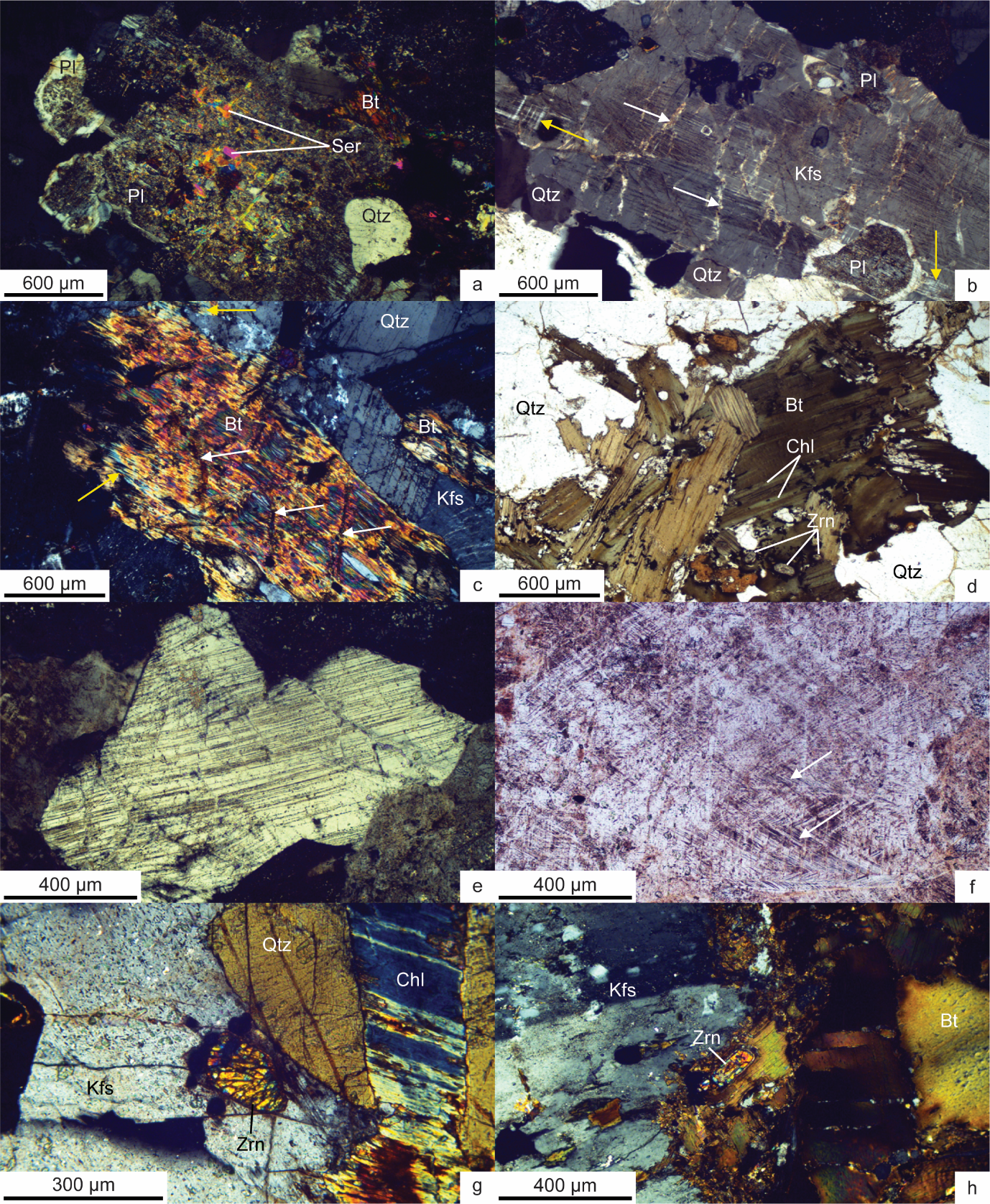


**Figure S1.** Cross-polarized (a-c, e, g, h) and linear-polarized (b, f) images of different mineral phases. (a) A strongly altered plagioclase with hetereogeneous, greyish to brownish interference colours. The interior of the grain is converted to sericite (sericitisation), a mineral phase with colourful interference colour of the third order. Two smaller plagioclase grains on the left side of the figure show fresh rims and strongly altered cores. The light grey mineral phase on the lower right side of the figure is quartz. (b) Large K-feldspar grain with perthitic exsolution lamellae (white arrows) and plagioclase inclusions suggesting a partly poikilitic texture of the larger host grain. Microcline twinning (yellow arrows) can be observed along the left rim and the lower right corner of the K-feldspar grain. (c) Shocked biotite with crossing kink bands (white arrows). This grain is partly altered to chlorite and thus, the colourful, second to third order interference colour of biotite is partly replaced by the anomalous bluish interference colour of chlorite, especially along the grain rim (yellow arrows). (d) Fresh biotite grains where some parts are greenish, indicative for alteration to chlorite. A few grains show a lath-shaped habits. In the lower middle part of the figure are zircons enclosed by biotite. The zircons are surrounded by a dark pleochroitic rim. (e) Shocked quartz with planar deformation features. (f) An altered K-feldspar grain showing ladder textures (see white arrows) in its lower right part, which are typically formed under the influence of shock pressure from an impact. (g) Zircon as an inclusion in K-feldspar. To the right is quartz with brownish to greyish interference colours and anomalous blue to bluish-yellow chlorite. Several subparallel fractures are generated within the zircon grain, suggesting a potential influence by shock pressure. (h) Metamict zircon with a strongly fractured, second to third order colourful rim and a core showing greyish-brownish interference colours. The grain is enclosed by biotite and to the left there is K-feldspar with grey interference colours.

**
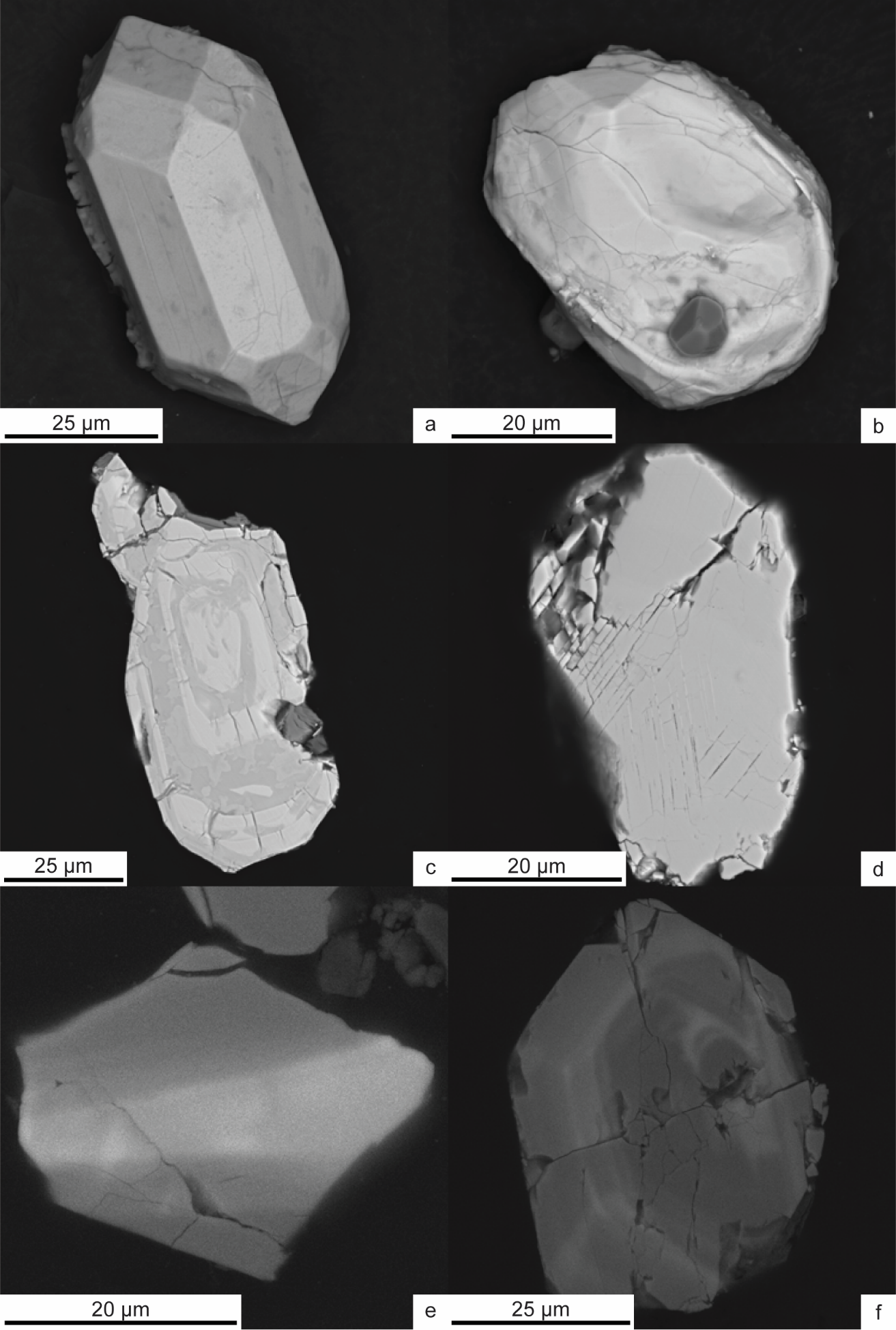
**

**Figure S2.** Back Scatter Electron (BSE)- (a-d) and Cathode Luminescence (CL) -derived (e, f) images of different, representative zircon textures observed in FE-SEM analyses. (a) Prismatic, euhedral-shaped zircon which are predominant in the samples. (b) Round-shaped zircon which can be rarely observed. (c) Metamict zircon showing oscillating zoning between non-metamict, fractured domains in light grey and metamict domains in dark grey. (d) Shocked zircon with two sets of parallel planar shock features which cross each other. (e) Zircon with an irregular CL pattern. (f) Zircon with oscillating zoning.


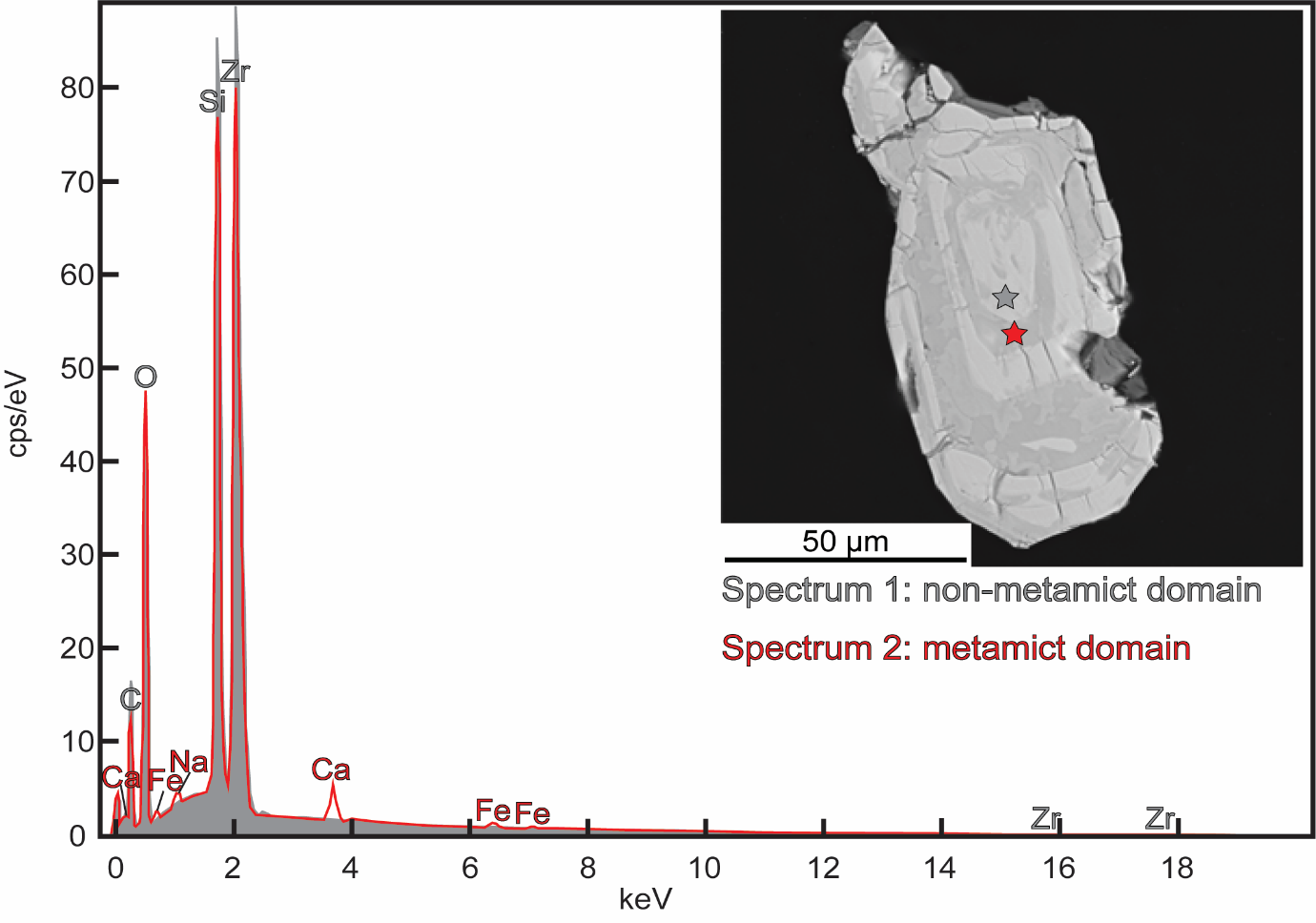


**Figure S3**. Qualitative element analyses of metamict zircon grain of Fig. S2c. Grey spectrum shows the elemental composition of the non-metamict, fractured, light grey domain. The red spectrum represents the metamict, dark grey domain with distinct peaks for Ca, Fe and Na. Previous studies have shown that solvent cations, e.g., Ca^2+^, Fe^2+^ and Na^+^, are often incorporated in metamict zircon grains in exchange of Pb^2+^, U^4+^, REE^3+^, Zr^4+^, Si^4+^, and Hf^4+^ to preserve the electron neutrality. The stars in the SE image mark the sites where both elemental spectra were recorded.


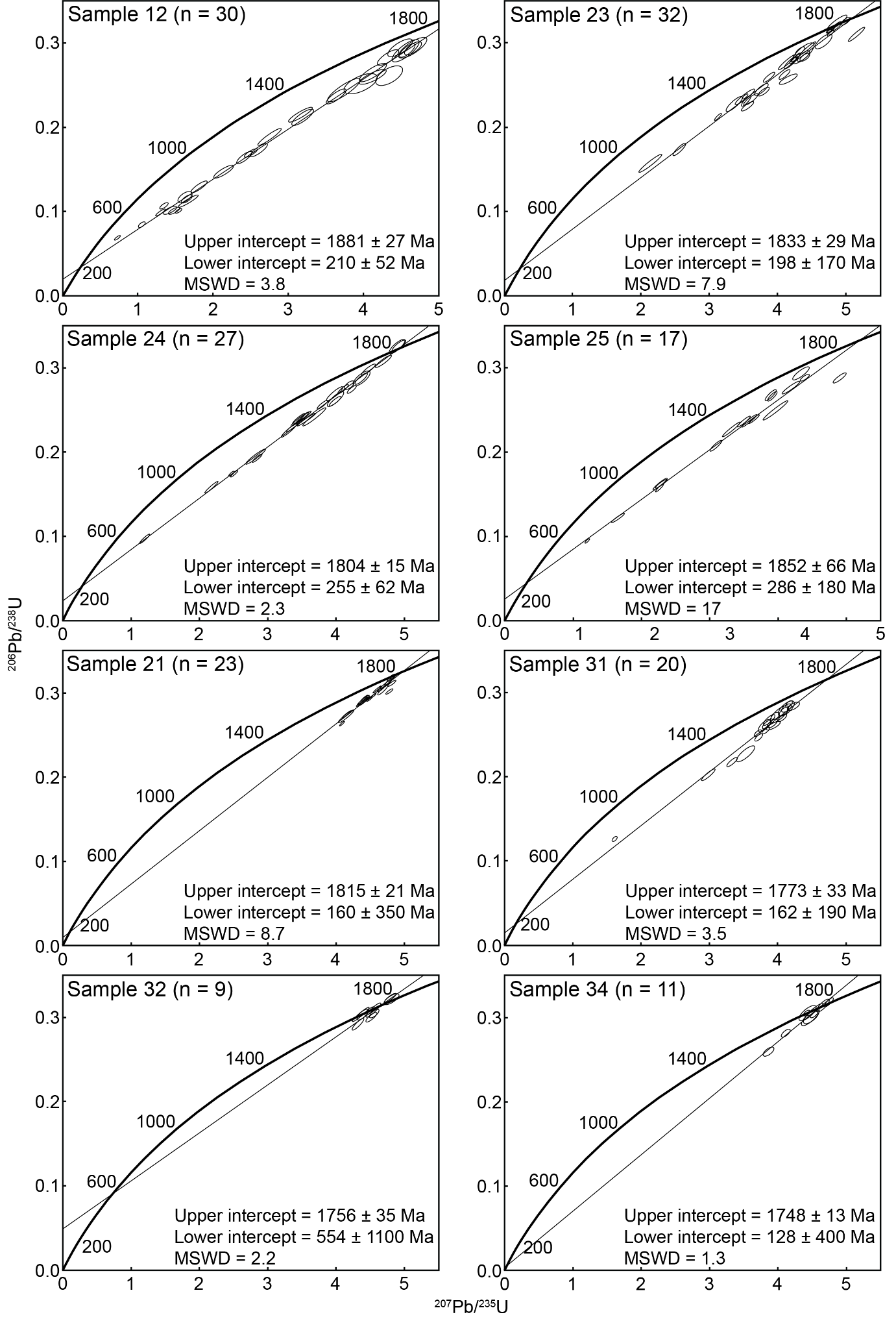


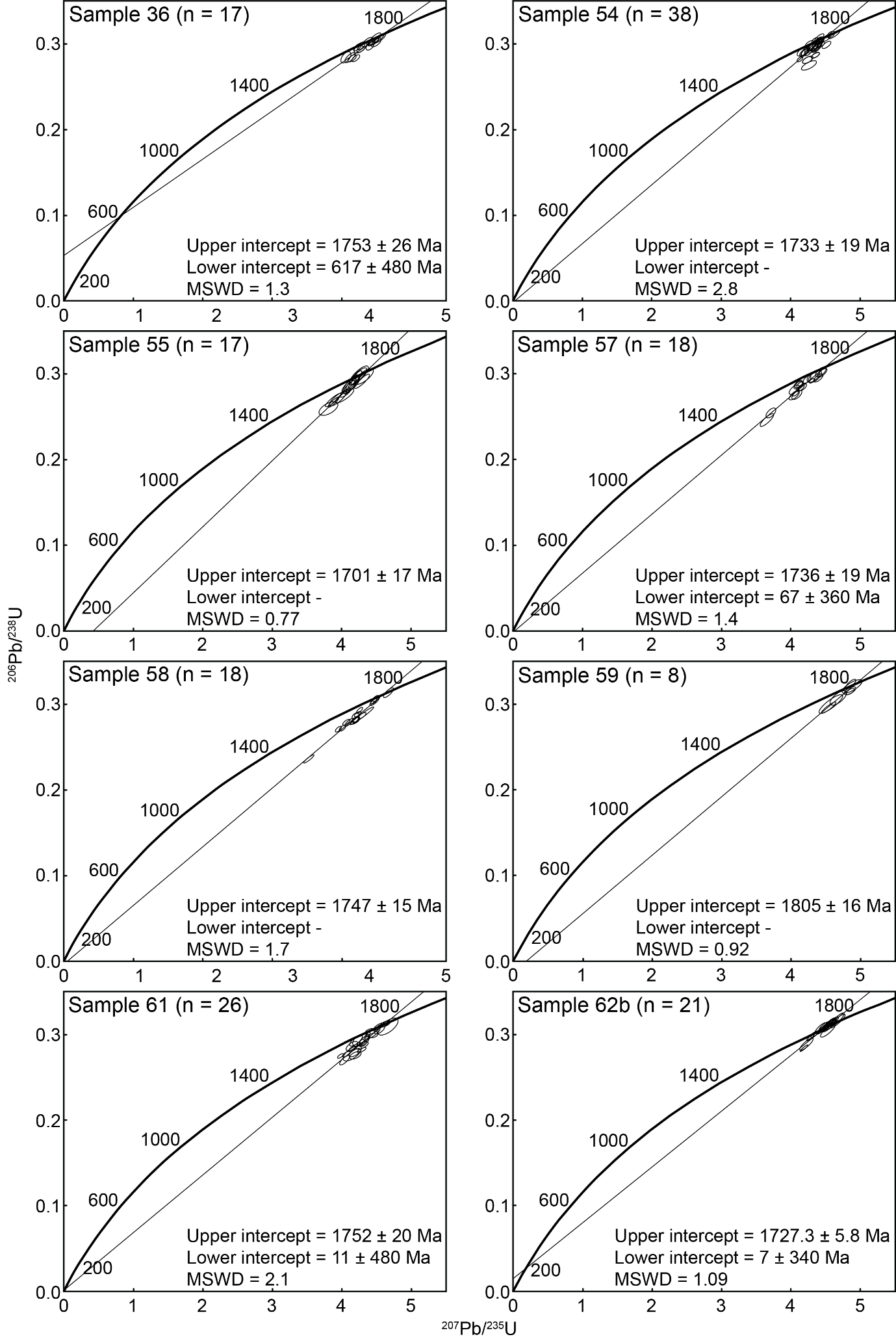


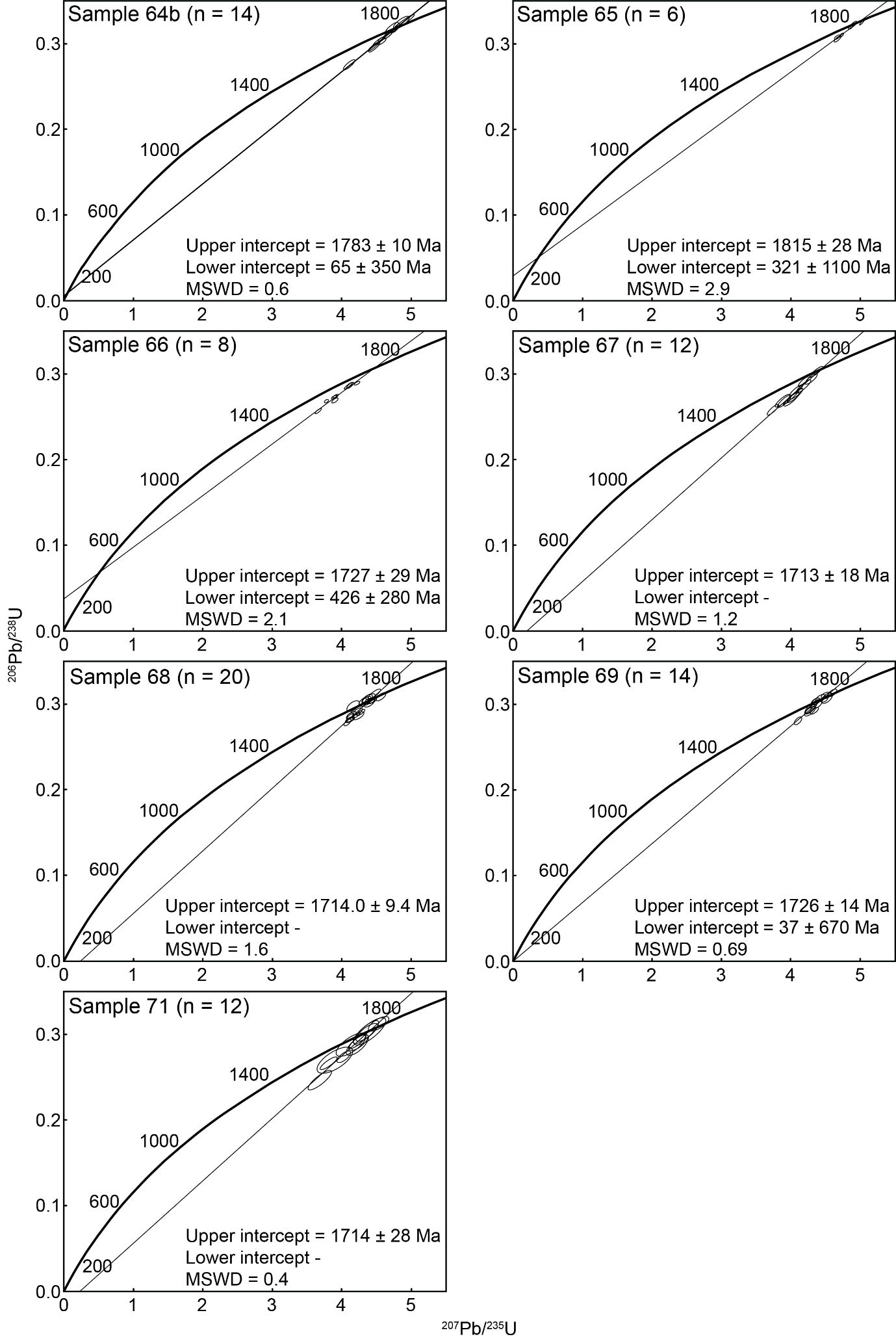


**Figure S4.** LA-ICP-MS U-Pb on zircon. The first 4 concordia plots (samples 12, 23, 24, and 25) are samples from outside from the central plateau, whereas the other plots showing the U-Pb data from samples taken from inside of the central plateau. The U-Pb analyses yield concordant to discordant data points plotting along the discordia line which forms two intercepts with the concordia curve. As clearly shown the U-Pb data of the samples from outside of the central plateau (samples 12, 23, 24, and 25) are generally more discordant than those from inside of the central uplift. The error ellipses are in 1σ uncertainties.


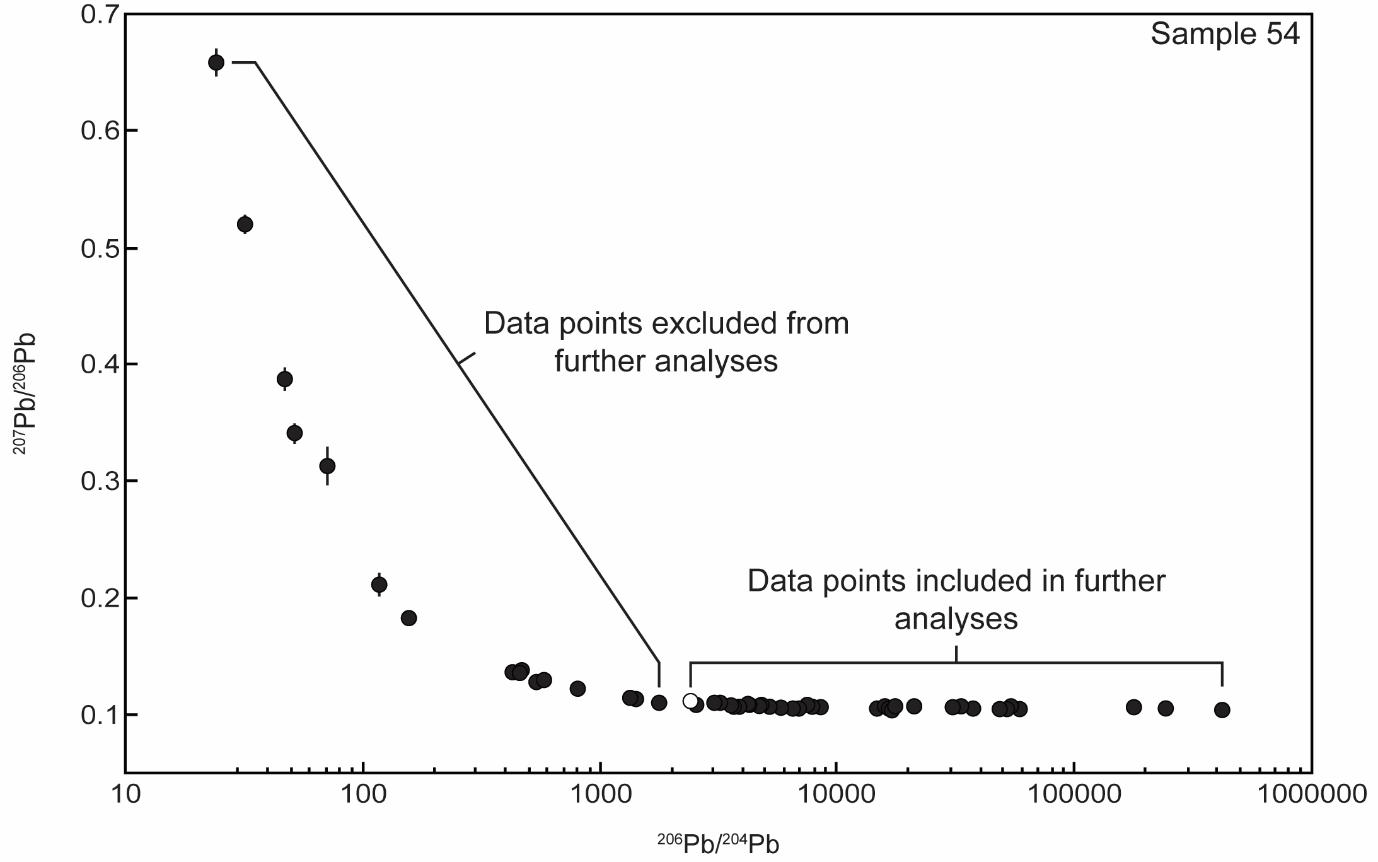


**Figure S5**. Plot ^206^Pb/^204^Pb vs ^207^Pb/^206^Pb on the example of sample 54. The white data point mark the threshold of ^206^Pb/^204^Pb = 2446 ± 108 set at that point where ^207^Pb/^206^Pb ratio show no changes. The threshold placed for the ^206^Pb/^204^Pb ratio varies between each sample, ranging mostly between 10^3^ and 10^4^.


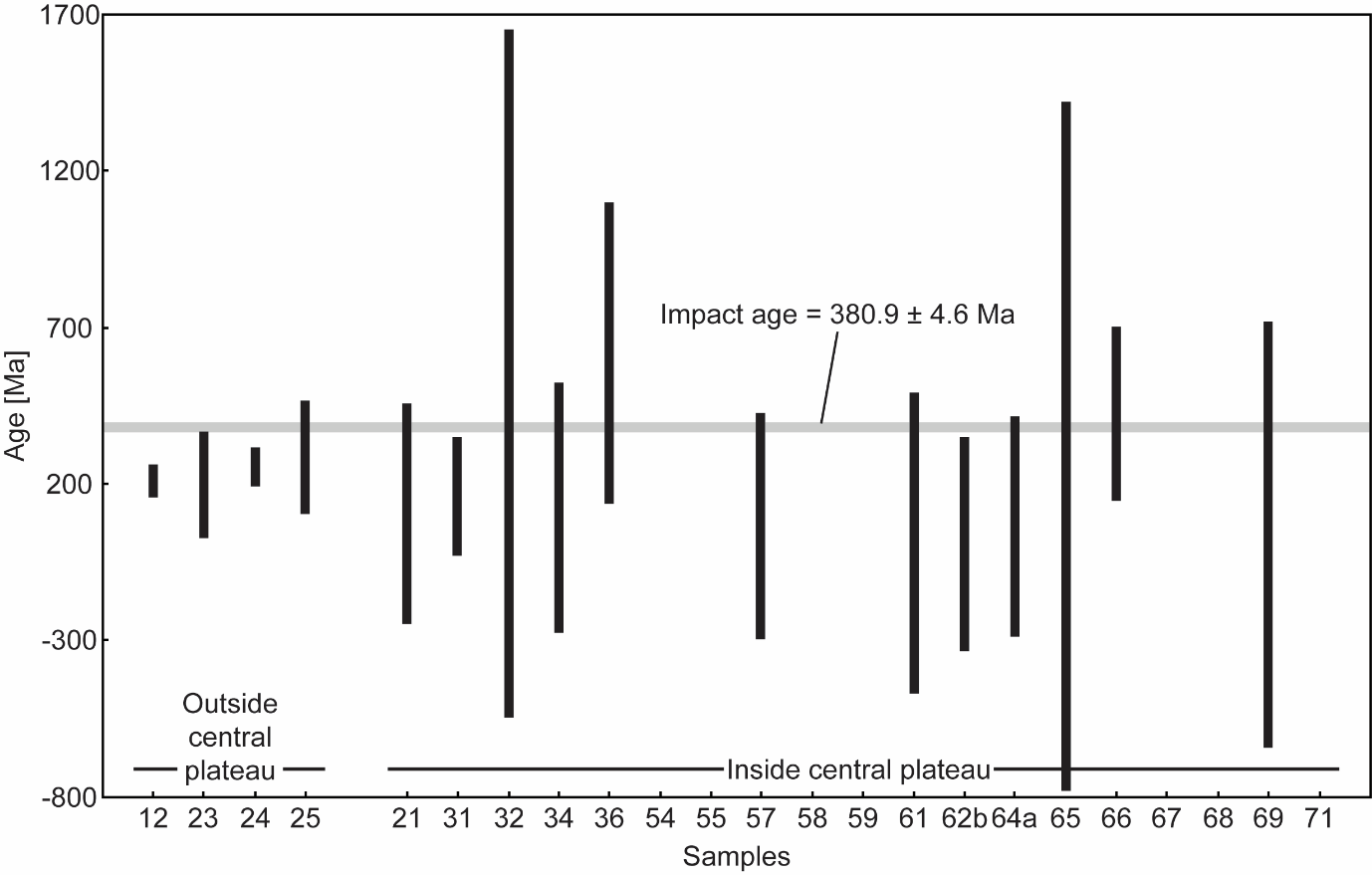


**Figure S6.** Lower intercept dates of zircon samples from outside (left side) and inside (right side) of the central plateau. There is no significant difference in the dates between outside and inside the central plateau. The known impact age = 380.9 ± 4.6 Ma^38^ (grey box) fall within the range of our lower intercepts.

**Table S1** with isotopic ratios and dates is given in a separate excel file. Isotopic ratios and dates of the primary and secondary reference material are given in **Table S2**, as separate excel file, as well. **Table S3** with mean discordance and number of analyses included to calculate the standard error and standard deviation for each eU concentration bin are given in a separate excel file. **Table S4** with LA-ICP-MS settings for U-Pb-Th dating is given as separate word file.
