# Supplementary material for: The effect of low-temperature annealing on discordance of U–Pb zircon ages
Source: Sci Rep. 2021 Mar 29;11:7079. doi: 10.1038/s41598-021-86449-y (PMC8007740; doi:10.1038/s41598-021-86449-y)
Supplement: Supplementary file 5 — Supplementary Information 5. [file 41598_2021_86449_MOESM5_ESM.docx]

**Table S4**. LA-ICP-MS settings for U-Th-Pb dating, Department of Geology, Lund University

| Laboratory & Sample preparation | |
| --- | --- |
| Laboratory name | LA-ICP-MS lab at Lund University |
| Sample type/mineral | Zircon |
| Sample preparation | Conventional mineral separation, 1 inch resin mount, 1 µm polish to finish |
| Imaging | BSE-imaging, CL-imaging, TESCAN Mira 3, 15 kV, 18 Na, 5-15 mm working distance |
| Laser ablation system |  |
| Make, Model & type | Photon machines, Analyte G2 excimer laser |
| Ablation cell & volume | HelEx 2 sample cell |
| Laser wave length | 193 nm |
| Pulse width | <4 ns |
| Fluence | 5-6 J/cm^-2^ |
| Repetition rate | 7-8 Hz |
| Spot size | 18x18 µm to 20x20 µm |
| Sampling mode/pattern | 400 µm^2^ single spot analyses |
| Carrier gas | He as carrier gas, Ar and N_2_ as make-up gas, combined down-stream sample chamber |
| Background correction | 25-30 seconds |
| Ablation duration | 30 seconds |
| Cell carrier gas flow | 0.8 l/min He and 6.5 ml/min N_2_ |
| ICP-MS Instrument | |
| Make, Model & type | Bruker Aurora Elite Quadrupole ICP-MS |
| Sample introduction | via 2 mm ID PFTE tubing with insert “squid” |
| RF power | ca. 1300 W |
| Make-up gas flow | ca. 0.95 l/min Ar |
| Detection system | Single collector discrete dynode electron multiplier or DDEM |
| Masses measured (dwell time in millisecond) | ^202^Hg (10), ^204^Pb (30-40), ^206^Pb (10), ^207^Pb (20-40), ^208^Pb (20-30), ^232^Th (10), ^238^U (10) |
| Total integration time per reading | ~1 sec |
| Total scan time | 120-150 milliseconds |
| Sensitivity | 20000 cps/ppm Pb |
| Dead time | 16 ns |
| Data processing | |
| Gas blank | 40 sec on peak |
| Calibration strategy | GJ-1 as primary and 91500 as secondary reference material |
| Reference material info | Wiedenbeck et al., (2004); Jackson et al., (2004) |
| Dataprocessing package used/Correction for LIEF | Iolite software (Paton et al., 2011), baseline correction and downhole correction |
| Mass discrimination | Standard-sample bracketing with ^207^Pb/^206^Pb and ^206^Pb/^238^U normalised to reference material GJ-1 |
| Common Pb correction, composition and uncertainty | No common Pb correction applied to the data |
| Uncertainty level | Ages are quoted at 2σ absolute |
| Quality control/Validation | GJ-1 Wtd ave ^206^Pb/^238^U age = 601.9 ± 0.2 Ma  (2σ, MSWD = 0.0023, n = 587)  91500 Wtd ave ^206^Pb/^238^U age = 1062.30 ± 0.97 Ma  (2σ, MSWD = 39.6, n = 148) |
